# Women's Experience of Continuity During Antenatal Care: A Cross‐Sectional Study in The Netherlands

**DOI:** 10.1111/birt.70008

**Published:** 2025-08-29

**Authors:** Evelien Cellissen, Marijke J. C. Hendrix, Luc Budé, Naaz Shareef, Maaike Vogels‐Broeke, Marianne J. Nieuwenhuijze

**Affiliations:** ^1^ Zuyd University Research Centre for Midwifery Science Maastricht the Netherlands; ^2^ Maastricht University Care and Public Health Research Institute (CAPHRI) Maastricht the Netherlands; ^3^ Radboud University Medical Center Faculty of Medical Sciences Nijmegen the Netherlands

**Keywords:** antenatal care, continuity of care, coordinating care professional, maternity care plan, midwifery, patient centered care, women's experiences

## Abstract

**Background:**

Continuity of maternity care contributes to improved birth experiences and health outcomes among women and newborns. To improve continuity of maternity care in the Netherlands, the Integrated maternity care standard recommends a maternity care plan and a coordinating care professional for all care settings. This study aimed to gain insights into women's experiences of continuity during antenatal care in the Netherlands in both community and hospital settings and whether a maternity care plan and coordinating care professional are associated with continuity of care as experienced by women.

**Methods:**

We conducted a cross‐sectional study in 2019 to 2020 among pregnant women (> 32 weeks) in the Netherlands. Experienced continuity of care was measured using the Nijmegen Continuity Questionnaire. We used regression analyses to explore the association between a maternity care plan, coordinating care professional, and experience of continuity during the antenatal period in multiple care settings.

**Results:**

We included 1165 women in this study. Most women reported moderate to high levels of continuity, with higher scores reported for care provided by community midwives compared to hospital staff. Having a maternity care plan, a coordinating care professional, and experiencing few care professionals were significantly associated with higher scores on continuity when care was provided by community midwives, but these associations were not found when care was provided by hospital staff.

**Discussion:**

Our findings emphasize the critical role of community midwives in promoting antenatal continuity. A maternity care plan, coordinating care professionals, and fewer care professionals contribute to this experience of continuity.

## Graphical abstract

In a study among 1,165 pregnant women in the Netherlands, continuity of antenatal care was rated higher for community midwives than for hospital staff. A maternity care plan, one coordinating professional, and fewer care professionals were linked to higher continuity scores for care by community midwives.

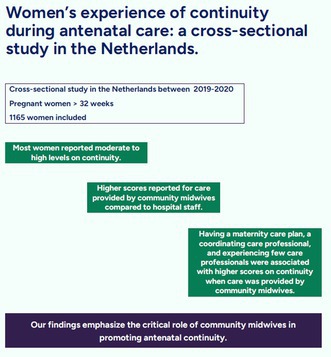

## 1 Introduction

Continuity of healthcare is a multidimensional concept and can be defined as patients' perceptions about their care coordination over time, including coherence, connection, and consistency in the care process. This involves interactions with one or more care professionals across different disciplines or organizations [1, 2, 3]. Continuity of care can be expressed in three dimensions. “Informational continuity” which implies that care professionals have access to the same information of patients' medical history and personal circumstances to tailor care to patients' needs. “Relational continuity” which is about building trust and a professional relationship between patients and care professionals, and “managerial continuity” which emphasizes well‐coordinated collaboration between professionals to ensure a connected and responsive care process [4, 5, 6]. Identifying and responding to these dimensions facilitates a patient‐centered approach, leading to improved health outcomes and patients' experiences [7, 8].

In maternity care, research shows that midwife continuity of care models improve perinatal outcomes and women's experiences [7, 9, 10, 11]. Several studies identified factors positively associated with continuity of maternity care, including the number of care professionals and the provision of care by community midwives [10, 11, 12]. However, most of these studies focus on women's experience of continuity throughout the whole perinatal period. Still, making sure that women specifically experience continuity during the antenatal period can help them to reveal relevant circumstances and better prepare for birth [13, 14]. Additionally, most research only focuses on midwife‐led care [9, 10, 12]. Accessibility to midwife‐led care varies across different countries, and women with medium‐ or high‐risk pregnancies currently often do not receive midwife‐led care [9, 10]. In the Netherlands, these women are referred from primary care, provided by community midwives, to secondary/tertiary care offered by obstetricians, obstetric registrars, hospital‐based midwives, and nurses, in this article further referred to as hospital staff. In 2019, 42% of the women in the Netherlands were referred during pregnancy. These women experienced an increasing number of care professionals and thereby discontinuity of care professional [15]. Therefore, continuity of care remains an ongoing concern in the Netherlands as well as in other countries, emphasizing the need to ensure and promote it.

In response, the Integrated maternity care standard was introduced to enhance continuity of care and improve maternal experiences [16]. Since 2016, all Dutch maternity care professionals are expected to implement this standard [17]. This standard contains key recommendations and organizational requirements for a safe, woman‐centered, Integrated maternity care system for all pregnant women. A maternity care plan, one of these recommendations, provides insights into the unique needs of each woman, thereby enabling care professionals to tailor their care to these needs. Furthermore, when women are referred from primary care to secondary/tertiary care, a maternity care plan facilitates communication between midwives, obstetricians, and women [5, 18, 19]. A coordinating care professional, another recommendation, ensures that care is tailored to the individual woman's needs and coordinates the efforts of all professionals involved [1, 20, 21]. Both key recommendations aimed at improving continuity of care as experienced by pregnant women in all care settings.

A better understanding about how women in different care settings experience continuity during antenatal care and whether continuity promoting recommendations from the care standard are associated with it will help to promote continuity of care. Currently, there is limited understanding of how women in the Netherlands experience continuity during antenatal care and to what extent the key recommendations from the Integrated maternity care standard: maternity care plan and coordinating care professional contribute to women's experience of continuity during antenatal care [1, 18]. Therefore, our study aims to explore:

- How pregnant women in the Netherlands experience continuity during antenatal care;
- The association between key recommendations from the Integrated maternity care standard and experience of continuity during antenatal care.

## 2 Methods

We used data from a cross‐sectional study among pregnant women in the Netherlands, collected between February 2019 and February 2020. Our study is part of a larger cross‐sectional study [StEM] [22] involving 3494 women during pregnancy (2nd and 3rd trimester) and postpartum. We analyzed the data of 1219 pregnant women in their 3rd trimester of pregnancy.

### 2.1 Participants

All midwifery practices and hospitals in the Netherlands were invited to participate. A total of 83 midwifery practices and 9 hospitals volunteered and participated, numbers that reflect the ratio of midwifery practices to hospitals in the Netherlands [15]. Care professionals invited pregnant women to participate, and additional recruitment was conducted via social media (Facebook, Twitter). Eligible participants were ≥ 32 weeks of pregnancy, > 18 years old, and proficient in Dutch. Women experiencing perinatal death or severe neonatal morbidity in their current pregnancy were excluded.

### 2.2 Data Collection

All women who agreed to participate received a questionnaire by post or email. If necessary, two reminders were sent after 1 week and after 3 weeks. Before initiating the questionnaire, all respondents signed an informed consent form. The Medical Ethics Committee Z, Heerlen   (registry number: METC‐Z‐20180121) approved the study. The questionnaire contained questions about women's background characteristics (age, educational level, parity, and nationality) and experienced number of care professionals with the response options: “very many,” “many,” “few,” and “very few.” We also asked women if they had a maternity care plan and a coordinating care professional, both with the response options: “yes,” “no,” and “Don't know.” For both questions, we presented the description mentioned in the Dutch Integrated maternity care standard [16] for clarity.

To explore women's experience of continuity during antenatal care, we used the Nijmegen Continuity Questionnaire (NCQ) [23], originally designed and validated to measure experience of continuity of care across multiple care settings [23, 24]. Since Dutch maternity care is organized across multiple settings, the NCQ captures the multidimensional nature of continuity, and it was used in other research as well, we selected it for our study [6, 11, 23, 24, 25]. Participants scored their experience of continuity during antenatal care on a 5‐point Likert scale (1 = strongly disagree, 5 (strongly agree) with higher scores indicating higher levels of experienced continuity [23]. The NCQ consisted of 28 items, across the following subscales:

- Personal continuity: care professional knows me (NCQ1), from both community midwives and hospital staff (5 items), concerning the care professional possesses knowledge about the woman and her medical history;
- Personal continuity: care professional shows commitment (NCQ2), from both community midwives and hospital staff (3 items)), emphasizing the care professional's commitment (3 items);
- Team continuity (NCQ3a), from both community midwives and hospital staff (4 items) about experienced cooperation between care professionals in the same care setting (4 items);
- Cross‐boundary continuity (NCQ3b), about experienced cooperation between care professionals in different care settings (4 items) [23].

We categorized women into three groups: (1) an unreferred group, including women who had up to the moment they filled out the questionnaire only received care from community midwives (unreferred, community midwives), (2) an unreferred group, including women who had up to the moment they filled out the questionnaire only received care from hospital staff (unreferred, hospital staff), and (3) a referred group, including women who had up to the moment they filled out the questionnaire received care from both community midwives and hospital staff (referred). Women in the group “unreferred, community midwives” filled out the NCQ 1, 2, 3a for community midwives. Women in the group “unreferred, hospital staff,” filled out the NCQ 1, 2, 3a for hospital staff, and women in the group “referred” filled out the NCQ 1, 2, 3a for both community midwives and hospital staff, and they filled in the NCQ3b for cross‐boundary continuity.

### 2.3 Statistical Analysis

Results for categorical variables are presented as frequencies and percentages; results for continuous variables are reported as means with standard deviations. We calculated the subscale scores on the NCQ as the mean of the item scores on each subscale [23]. We excluded participants with one or more missing values within a subscale. Differences between means per NCQ subscale within the referred groups and within the unreferred groups were tested with a t‐test. Differences between the three groups concerning frequencies and percentages for maternity care plan, coordinating care professional, and experienced number of care professionals were tested with the Chi‐square test. A *p* ≤ 0.05 was considered statistically significant.

For the items “maternity care plan” and “coordinating care professional”, we recoded the answer option “Don't know” into “no”. We did that under the assumption that women who are not sure about a maternity care plan were unlikely to have actively engaged in it, and women who are not sure about a coordinating care professional were unlikely to have had one. For the item “experienced number of care professionals”, we recoded the answer options “very many” and “many” into “many,” and “few” and “very few” into “few,” yielding a dichotomous variable. We conducted sensitivity analyses to assess the robustness of our results by comparing the original and recoded data.

As a result of an error in the questionnaire design, women were given the possibility to answer “Don't know” on the three NCQ subscales. We recoded these answers to “moderate” (“3” on the five‐point Likert scale). We conducted sensitivity analyses to assess the robustness of our results by comparing the original and recoded data. On NCQ3a we gave women the opportunity to fill in the answer “not applicable” for women who received all care up to then in a caseload midwifery practice with only one care professional. We excluded these cases from the analysis of NCQ subscale 3a.

To explore factors associated with the experience of continuity of care, we performed separate regression analyses per group. For this purpose, dependent variables were created for each subscale and all three groups (Table 1).

**TABLE 1:** Dependent variables for regression analyses.

Unreferred, community midwives	Unreferred, hospital staff	Referred
NCQ1_unreferred_communitymidwives_	NCQ1_unreferred_hospitalstaff_	NCQ1_referred_communitymidwives_
NCQ2_unreferred_vcommunitymidwives_	NCQ2_unreferred_hospitalstaff_	NCQ2_referred_communitymidwives_
NCQ3a_unreferred_communitymidwives_	NCQ3a_unreferred_hospitalstaff_	NCQ3a_referred_communitymidwives_
		NCQ1_referred_hospitalstaff_
		NCQ2_referred_hospitalstaff_
		NCQ3a_referred_hospitalstaff_
		NCQ3b_referred_

*Note:* NCQ1 = personal continuity: care professional knows me; NCQ2 = personal continuity: care professional shows commitment; NCQ3a = team continuity; NCQ3b = cross‐boundary continuity.

Abbreviation: NCQ, Nijmegen Continuity Questionnaire.

As independent variables, we used maternity care plan and coordinating care professional. Additionally, several potential confounding variables were identified as they might affect the experienced continuity of care, including the variables experienced number of care professionals, age, educational level, parity, and nationality [12, 26, 27, 28, 29, 30]. These variables were included in the regression model. Categorical variables with more than two levels were recoded into dummy variables. Missing values were designated “system missing” and we excluded these cases from analysis. We conducted a backward‐selection strategy to refine the regression model by iteratively removing non‐significant variables until only statistically significant predictors remained. A *p* ≤ 0.05 was considered statistically significant. We analyzed the data using SPSS version 29.0.

## 3 Results

We distributed the questionnaire among 1652 women who were at least 32 weeks pregnant; 1219 returned the questionnaire (response rate 73.8%). Because of missing values, we excluded 54 questionnaires from the analyses, leaving 1165 included responses. Among participants, 706 women (60.6%) received primary care from midwives (unreferred), 45 women (3.9%) received secondary/tertiary care from hospital staff (unreferred), and 414 women (35.5%) received both primary care from midwives and secondary/tertiary care from hospital staff (referred). The latter group was referred from primary to secondary/tertiary care during pregnancy (Table 2).

**TABLE 2:** Characteristics of the participants.

Characteristics	Total (*n* = 1.165)		Unreferred community midwives (*n* = 706)		Unreferred hospital staff (*n* = 45)		Referred community midwives and hospital staff (*n* = 414)	
	*n*	(%)	*n*	(%)	*n*	(%)	*n*	(%)
Age in years	Mean 30.5		Mean 30.1		Mean 33.2		Mean 30.8	
Educational level								
Low	56	(4.8)	37	(5.2)	2	(4.4)	17	(4.1)
Middle	427	(36.7)	267	(37.8)	15	(33.3)	145	(35)
High	682	(58.5)	402	(56.9)	28	(62.2)	252	(60.9)
Parity								
Nulliparous	439	(37.7)	277	(39.2)	7	(15.6)	155	(37.4)
Multiparous	726	(62.3)	429	(60.8)	38	(84.4)	259	(62.6)
Nationality								
Dutch	1035	(89.8)	632	(89.5)	37	(82.2)	366	(88.4)
Non‐Dutch	130	(11.2)	74	(10.5)	8	(17.8)	48	(11.6)

In Table 3 the mean scores on the three subscales of the NCQ are presented per group. Women reported higher mean scores on continuity of care on all three subscales when receiving care from community midwives, compared to hospital staff (*p* < 0.001). This applies to both referred and unreferred women. All groups reported higher mean scores on team continuity (NCQ3a) compared to personal continuity (NCQ1 and NCQ2).

**TABLE 3:** Experience of continuity during antenatal care (NCQ).

	Unreferred (*n* = 751)			Referred (*n* = 414)		
	Community midwives (*n* = 706)	Hospital staff (*n* = 45)		Community midwives (*n* = 414)	Hospital staff (*n* = 414)	
	Mean (SD)	Mean (SD)		Mean (SD)	Mean (SD)	
NCQ (scoring range 1–5)						
Personal continuity, care professional knows me (NCQ1)	3.74 (0.72)	3.24 (0.79)	(*p* < 0.001)	3.66 (0.76)	2.78 (0.72)	(*p* < 0.001)
Personal continuity, care professional shows commitment (NCQ2)	3.61 (0.66)	2.99 (0.82)	(*p* < 0.001)	3.75 (0.76)	2.86 (0.78)	(*p* < 0.001)
Team continuity (NCQ3a)	4.17 (0.69)*	3.48 (0.87)	(*p* < 0.001)	4.14 (0.72)**	3.42 (0.70)	(*p* < 0.001)
Cross‐boundary continuity (NCQ3b)	n.a.	n.a.		3.55 (0.70)		

*Note:* This table presents mean scores (with SD) on the three NCQ subscales representing women's experience of continuity during antenatal care. *p* < 0.05 = statistically significant.

Abbreviation: n.a., not applicable.

* *n* = 619.

** *n* = 392.

Of all women, 264 women (22.7%) responded that they had a maternity care plan, and 502 women (43.1%) responded they had a coordinating care professional (Table 4). There was no statistically significant difference for maternity care plan (*p* = 0.897) and coordinating care professional (*p* = 0.074) between the groups unreferred community midwives, unreferred hospital staff, and referred. The sensitivity analysis for experienced number of care professionals showed no different results between original data and recoded data. Neither showed the sensitivity analysis different results for the three NCQ subscales.

**TABLE 4:** Having a maternity care plan, a coordinating care professional, and experienced number of care professionals.

	Community midwives (*n* = 706)		Hospital staff (*n* = 45)		Both midwives and hospital staff (*n* = 414)		
	*n*	(%)	*n*	(%)	*n*	(%)	
Having a maternity care plan (yes)	157	(22.2)	10	(22.2)	97	(23.4)	(*p* = 0.897)
Having a coordinating care professional (yes)	286	(40.5)	23	(51.1)	193	(46.6)	(*p* = 0.074)
Experienced number of care professionals							
Few	515	(72.9)	17	(37.8)	207	(50)	(*p* < 0.001)
Many	191	(27.1)	28	(62.2)	207	(50)	(*p* < 0.001)

*Note:* This table presents the frequencies (%) of women who reported having a maternity care plan, a coordinating care professional, and a few or many care professionals.

### 3.1 Factors Associated With Experience of Continuity During Antenatal Care

Tables 5 and 6 present the independent variables associated with the experience of continuity during antenatal care provided by community midwives. The dependent variables, reflecting the experience of continuity, are represented by the NCQ subscales NCQ1, NCQ2, and NCQ3a.

**TABLE 5:** Results from the regression analyses for the group “unreferred, community midwives”: Variables associated with experience of continuity during antenatal care (NCQ).

	NCQ1_unreferred_communitymidwives_				NCQ2_unreferred_communitymidwives_				NCQ3a_unreferred_communitymidwives_			
	Unstandard *β*	Standard *β*	*p*	CI	Unstandard *β*	Standard *β*	*p*	CI	Unstandard *β*	Standard *β*	*p*	CI
Maternity care plan			n.s.		0.233	0.146	< 0.001	[0.123; 0.344]			n.s.	
Coordinating care professional	0.384	0.260	< 0.001	[0.281; 0.486]	0.343	0.254	< 0.001	[0.248; 0.438]	0.224	0.156	< 0.001	[0.113; 0.335]
Experienced number of care professionals												
Many	Reference				Reference				Reference			
Few	0.342	0.210	< 0.001	[0.228; 0.457]	0.222	0.149	< 0.001	[0.118; 0.327]	0.226	0.150	< 0.001	[0.109; 0.343]
Educational level												
Low	Reference				Reference				Reference			
Middle	−0.261	−0.175	0.026	[−0.492; −0.031]			n.s.		−0.289	−0.204	0.019	[−0.530; −0.048]
High	−0.283	−0.194	0.014	[−0.510; −0.057]			n.s.		−0.276	−0.200	0.022	[−0.513; −0.040]
Parity												
Nulliparous	Reference				Reference				Reference			
Multiparous	0.132	0.089	0.012	[0.030; 0.235]	0.133	0.048	0.005	[0.039; 0.227]			n.s.	
		*R* ^2^ = 0.158				*R* ^2^ = 0.151				*R* ^2^ = 0.060		

*Note:* NCQ1 = personal continuity: care professional knows me, NCQ2 = personal continuity: care professional shows commitment, NCQ3a = team continuity.

Abbreviation: n.s., not statistically significant.

^a^ The variables age and nationality were removed from the regression model as they were not statistically significant.

**TABLE 6:** Results from the regression analyses for the group “referred, community midwives”: Variables associated with experience of continuity during antenatal care (NCQ).

	NCQ1_referred_communitymidwives_				NCQ2_referred_communitymidwives_				NCQ3a_referred_communitymidwives_			
	Unstandard *β*	Standard *β*	*p*	CI	Unstandard *β*	Standard *β*	*p*	CI	Unstandard *β*	Standard *β*	*p*	CI
Maternity care plan	0.298	0.165	< 0.001	[0.133;0.463]	0.257	0.144	0.003	[0.087; 0.427]	0.262	0.154	0.002	[0.097; 0.427]
Coordinating care professional	0.215	0.141	0.003	[0.073; 0.357]	0.200	0.132	0.008	[0.054; 0.346]			n.s.	
Experienced number of care professionals												
Many	Reference				Reference				Reference			
Few	0.368	0.241	< 0.001	[0.228; 0.508]	0.255	0.168	< 0.001	[0.110; 0.399]	0.338	0.235	< 0.001	[0.199; 0.476]
Parity												
Nulliparous	Reference											
Multiparous	0.235	0.149	0.001	[0.093; 0.377]			n.s.				n.s.	
		*R* ^2^ = 0.147				*R* ^2^ = 0.076				*R* ^2^ = 0.071		

*Note:* NCQ1 = personal continuity: care professional knows me, NCQ2 = personal continuity: care professional shows commitment, NCQ3a = team continuity.

Abbreviation: n.s., not statistically significant.

^a^ The variables age, nationality, and educational level were removed from the regression model as they were not statistically significant.

### 3.2 Unreferred, Community Midwives

Table 5 shows the results of the regression analysis for unreferred women who received primary care from midwives. Having a maternity care plan contributed to higher scores on personal continuity (NCQ2). Having a coordinating care professional and experiencing fewer care professionals contributed to higher scores on personal continuity (NCQ1, NCQ2) and team continuity (NCQ3a) (Table 5).

### 3.3 Referred, Community Midwives

Table 6 presents the results of the regression analysis for referred women regarding antenatal care they received from community midwives. Having a maternity care plan and experiencing fewer care professionals were associated with higher scores on the three NCQ subscales. Having a coordinating care professional contributed to higher scores on personal continuity (NCQ1, NCQ2) (Table 6).

### 3.4 Unreferred and Referred, Hospital Staff

In unreferred women scoring care from hospital staff, a negative association was found between personal continuity (NCQ2) and low educational level compared to middle and high (not in a table). In this group, no other associations were found. In referred women scoring continuity during antenatal care from hospital staff, no associations were found.

## 4 Discussion

In this study, we explored how pregnant women in the Netherlands experience continuity during antenatal care when receiving care from community midwives or hospital staff. Most women experienced moderate to high levels of continuity, with higher levels reported for care provided by community midwives compared to hospital staff. This applied to both referred and unreferred groups. Additionally, we explored if key recommendations from the Integrated maternity care standard to promote continuity of care were associated with the experience of continuity during antenatal care. Having a maternity care plan, a coordinating care professional, and experiencing few care professionals were significantly associated with higher levels of continuity when care was provided by community midwives, but these associations were not found for care provided by hospital staff.

Our findings demonstrated that women who received care from community midwives, within the referred and within the unreferred group, reported statistically significant higher mean scores on both personal (NCQ1 and 2) and team continuity (NCQ3a) compared to women who received care from hospital staff. This result is consistent with the NCQ scores reported in a similar study [11] and prior research showing that midwife‐led care contributes to women's experienced continuity of care [7, 10]. Some studies have indicated that, in addition to midwife‐led care, experiencing care from fewer care professionals also contributes to continuity of care [10, 11, 12]. In our study, the variable experienced number of care professionals partly explains the higher scores on experienced continuity from community midwives, as shown in our regression analyses. We found that women who experienced fewer care professionals reported higher scores on both personal and team continuity of care from community midwives. One mechanism might be that it is easier for women to build personal relationships with a few care professionals rather than many, as they see the same person more often. These personal relationships enable care professionals to better understand women's preferences, an important aspect of how women experience continuity of care [13]. Another possible explanation for the reported higher scores on continuity from community midwives compared to hospital staff might be the multidisciplinary nature of hospital staff, as also suggested by Perdok et al. [11] This diverse staff composition might lead to a lack of awareness of women's preferences and discontinuity in information women receive, both of which are elements that contribute to experienced continuity of care [4, 24, 31, 32].

Our study confirmed previous findings that team continuity was scored higher than personal continuity [11]. This might be attributed to an increased emphasis in Dutch maternity care on enhancing collaboration among maternity care professionals, guided by the Integrated maternity care standard [16, 33]. This focus on collaboration within and between maternity care organizations seems to contribute to higher levels of experienced team continuity. However, our study revealed moderate levels for cross‐boundary continuity, suggesting room for improvement in inter‐organizational collaboration.

As continuity of care improves women's experiences, we aim for high levels of experienced continuity for all women, regardless of the care professional or referral status [7, 9, 10, 11]. Although the exact clinically meaningful cut‐off scores on the NCQ subscales are not known, it seems acceptable that continuity scores above “3 (moderate)” are a minimal aim. Our results show that there is more room for improvement in continuity of care from hospital staff compared to care from community midwives. It appears that other factors, beyond those we have explored, influence the experience of continuity during antenatal care from hospital staff. Similarly, for community midwives, the *R* ^2^ value from the regression analyses indicates that additional, unexplored factors are associated with the experience of continuity during antenatal care. Other research suggests that factors like personal preferences, pregnancy history, status, and organizational or communication aspects may influence continuity of care for both community midwives and hospital staff [13, 34]. Further research is needed to explore their potential role in shaping experienced continuity of care.

In our study, only a minority of women had a maternity care plan or a coordinating care professional, despite recommendations in the Integrated maternity care standard. This finding is consistent with policy evaluations and other research results [17, 35]. We found an association between having a maternity care plan and higher levels of experienced continuity when care was provided by community midwives. This might be because a maternity care plan facilitates information exchange between professionals and women, enhancing personal continuity [18, 19]. Additionally, a maternity care plan enables tailored care, fostering familiarity and personal attention, which are essential for continuity of care [13, 36]. It also promotes effective communication among professionals, allowing them to synchronize efforts and share appointments, thereby ultimately improving the woman's perception of coordinated care and contributing to team continuity [32]. Surprisingly, we found no association between having a maternity care plan or a coordinating care professional and experience of continuity during antenatal care from hospital staff. For the unreferred women, cared for by hospital staff, this might be explained by the small group size (*n* = 45). For the referred group, we cannot explain these differences in associations between community midwives and hospital staff from our results, but it might be due to variations in content and utilization of maternity care plans or coordinating care professionals by different professionals as described in other research, potentially leading to diverse impacts on experienced continuity of care [18, 35]. These variations may arise from differences in how maternity care plans and coordinating care professionals are implemented in practice. While the Integrated maternity care standard may have supported community midwives, it may not have effectively addressed hospital staff needs or motivation. To enhance continuity of care, policies must align more closely with practical realities, ensuring clarity in content and consistency across both community and hospital settings [37, 38]. Since we do not know whether this is the case, further research is needed to identify barriers and facilitators among healthcare professionals regarding the implementation of maternity care plans and a coordinating care professional. Promoting these recommendations for all pregnant women is essential, as nearly all women receive care from a community midwife, regardless of referral status.

## 5 Strengths and Limitations

Our results were based on a large sample of 1165 pregnant women spread throughout the Netherlands with a high response rate of 74%. While our sample mostly mirrored the general pregnancy population in the Netherlands, there is a small overrepresentation of ethnic Dutch women (89.8%) compared to the national pregnant population (86.3%) [15] and highly educated women (58.5%) compared to the national pregnant population (53.7%) [15] (Table 2). The exclusive availability of the questionnaire in Dutch may have inadvertently excluded women with limited proficiency in the language. A strength of our study is its novelty in quantitatively examining continuity of care using the NCQ, as continuity of care is often explored qualitatively [39], though this also means there is limited research for comparison [39]. Continuity of care is a multidimensional concept and our questionnaire did not apparently capture relevant factors associated with the experience of continuity from hospital staff. For referred women, a possible explanation for the differences in experienced continuity of care between community midwives and hospital staff might be due to the timing of referral. As most referrals during pregnancy occur in the third trimester [15], women in this group might mostly be cared for by community midwives and subsequently experienced less (continuity of) care from hospital staff. Still, our study provides valuable insights into continuity of care during the antenatal period in the Netherlands.

## 6 Conclusion

Most women in our study seem to experience moderate to high levels of continuity during antenatal care, higher among women receiving care from community midwives compared to hospital staff (with or without referral). This underscores the role of midwives in contributing to continuity of care. Having a maternity care plan, a coordinating care professional, and seeing fewer care professionals significantly contributed to the experience of continuity from community midwives (with or without referral). No such associations were found for hospital staff, needing further exploration with larger samples. As nearly all women, referred or not, receive midwifery care during pregnancy, we recommend promoting the use of maternity care plans, coordinating care professionals, and limiting the number of care professionals to enhance the experience of continuity during antenatal care.

## Conflicts of Interest

The authors declare no conflicts of interest.

## Data Availability

The data that support the findings of this study are available from the corresponding author upon reasonable request.
